# Analysis of Risk Factors and Nursing Strategies for Unplanned Extubation in Children: Retrospective Cohort Study

**DOI:** 10.2196/71307

**Published:** 2025-06-10

**Authors:** Xuefeng Han, Hairong Liu, Tingchong Zhang, Guangxin Fan

**Affiliations:** 1 Pediatric Surgery, Shunyi District Women and Children's Hospital, Beijing Children’s Hospital, Beijing, China; 2 Shunyi Maternity and Child Hospital, Beijing Children's Hospital, Capital Medical University, Beijing, China; 3 Pediatric Surgery, Beijing Children’s Hospital, Capital Medical University, No.56 Nanlishi Road Xicheng District, Beijing, China, 86 13370115122

**Keywords:** unplanned extubation, nursing strategies, prevention, risk factor, pediatric care

## Abstract

**Background:**

Unplanned extubation (UEX) is a critical indicator of nursing care quality. Existing research primarily focuses on pediatric intensive care units (PICUs), with limited data available from general pediatric surgery. Currently, most studies on this topic are mainly focused on PICUs, and there is a lack of research data regarding general pediatric surgery. Therefore, further research should be conducted based on this consideration.

**Objective:**

This study aimed to analyze the high-risk factors for UEX in children and implement appropriate nursing strategies to reduce its incidence, ensuring clinical safety of pediatric patients.

**Methods:**

A retrospective study (January 2018 - December 2023) included pediatric patients with indwelling catheters in general surgery. Exclusion criteria included mental disorders or abnormal Glasgow Coma Scale scores. Data on catheter days, UEX incidents, and risk factors were analyzed.

**Results:**

A total of 1977 catheter days were recorded during the perioperative period, comprising 1079 days with urinary catheters, 768 days with postoperative wound drainage tubes, 68 days with gastric tubes, 46 days with peripheral central venous catheters, and 8 days with central venous catheters. During this period, 13 incidents of UEX occurred, yielding an overall UEX rate of 6.58 per 1000 catheter days. Urinary catheters accounted for the highest proportion of UEX incidents (8/13, 61.5%), followed by gastric tubes (3/13, 21.3%) and postoperative wound drainage tubes (2/13, 15.4%). The reintubation rate following UEX was 15.38% (2/13). Further analysis identified significant risk factors associated with UEX: (1) patient characteristics: age ≤3 years (8/13, 61.5%) and male sex (10/13, 76.9%); (2) clinical management: absence of physical restraints (10/13, 76.9%); and (3) temporal factors: incidents occurring during holidays (9/13, 69.2%). Multivariate analysis revealed that UEX risk was influenced by inter-related factors, including pediatric physiological characteristics (eg, limited self-regulation capacity), suboptimal catheter fixation methods, positional discomfort during patient movement, and variations in nursing interventions (eg, frequency of rounds and caregiver education).

**Conclusions:**

Unplanned extubation in pediatric inpatients represents a critical clinical complication that may compromise treatment efficacy and prolong hospitalization. Our findings highlight the multifactorial etiology of UEX events, with risk determinants spanning patient characteristics, care protocols, and environmental factors. To mitigate these risks, we propose implementing evidence-based multidisciplinary preventive strategies, including standardized risk assessment protocols for high-risk subgroups (eg, male patients aged ≤3 years), enhanced staff training on age-appropriate restraint techniques and securement device utilization, and dynamic adjustment of nursing surveillance frequency during peak risk periods (eg, holidays or postural changes). This systematic approach demonstrates potential to reduce UEX-associated adverse events by 42%‐68%, according to benchmark studies, ultimately improving pediatric care quality.

## Introduction

### Unplanned extubation

Unplanned extubation (UEX), a critical patient safety indicator reflecting nursing care quality, can lead to irreversible clinical consequences across all age groups. In adult critical care settings, reported UEX incidence ranges from 7% to 18%, with endotracheal tube dislodgement demonstrating particularly severe outcomes at rates of 0.2%‐14.6% [1-3]. The UEX rate for indwelling nasogastric tubes was 7.6% (95/1243) [4]. A total of 56,508 courses occurred in 36,696 patients, with a crude UEX rate of 2.8% [5], and 0.43‐0.79% in pediatric intensive care units (PICUs) [6]. The clinical implications of pediatric UEX extend beyond immediate physiological risks. When delayed reintubation occurs, these events may disrupt therapeutic trajectories, prolong hospitalization duration by 2‐5 days based on severity [7], exacerbate family-caregiver tensions, and ultimately degrade health care service quality metrics. Despite these consequences, UEX in pediatric general surgical contexts remains understudied. To address this knowledge gap, we conducted a retrospective cohort analysis of UEX incidents recorded in a tertiary pediatric general surgery department between January 2018 and December 2023 (N=1977 catheter days). Through multidimensional risk factor identification, this study provides actionable evidence for developing nurse training protocols targeting high-risk scenarios, optimizing hospital administration policies for device securement, implementing preventive bundles tailored to pediatric surgical populations.

### Definition and Classification of UEX

Unplanned extubation (UEX) is formally defined as the premature removal of indwelling medical devices prior to therapeutic protocol completion, occurring through: (1) Self-extubation: patient-initiated device removal without clinical authorization; (2) accidental dislodgement: unintentional tube slippage due to iatrogenic factors (eg, improper securement during nursing procedures); and (3) therapeutic failure: early device removal mandated by compromised functionality (eg, obstruction leakage or material degradation) [8].

This tripartite classification system emphasizes UEX causation mechanisms, facilitating targeted prevention strategies across clinical scenarios.

The incidence rate of UEX can be calculated in two ways [5,9]: (1) number of UEX instances of a particular catheter during a specific period/total days of catheter placement during the study period × 1000‰, (2) number of UEX instances of a particular catheter/total number of instances of catheter placement during the study period × 1000‰. The first method is more commonly used, particularly in monitoring tracheal intubation UEX in intensive care units (ICUs). This method was also used in monitoring catheter infection rates during the 2011 assessment of tertiary comprehensive hospitals. This study adopts the first method for calculating incidence.

## Methods

### Study Setting

This retrospective study enrolled pediatric patients (aged <18 years) who: (1) underwent general surgical procedures with indwelling catheters between January 2018 and December 2023; (2) demonstrated preserved neuromuscular integrity (ie, normal muscle tone, Glasgow Coma Scale (GCS) ≥14); and (3) had no documented neuropsychiatric comorbidities affecting device tolerance.

Catheter types analyzed included preoperative peripherally inserted central catheters and intraoperative or postoperative devices such as gastric tubes, urinary catheters, central venous catheters, and surgical wound drainage systems. Patient demographics, catheter maintenance characteristics, and contextual factors during UEX events were systematically documented through electronic health record extraction.

### Research Method

This study used a retrospective analysis approach for UEX. According to the hospital protocol, UEXs were reported as adverse events to the nursing department using a hospital-specific reporting form. The types of tubes included, in addition to those previously mentioned, were endotracheal tubes, tracheostomy tubes, fistula tubes, arterial lines, and dialysis catheters. However, as our department does not use these additional tubes, they were not included in this study. The Richmond Agitation-Sedation Scale (RASS) and the pediatric-modified GCS were applied for assessment. The RASS is a commonly used tool in health care to assess the level of sedation and agitation in patients and can provide guidance to nurses. The RASS-specific scoring criteria are presented in Table 1 [10].

**Table 1.** The RASS scoring criteria [11].

Score	Status	Clinical symptoms
+4	Aggressive	Violence
+3	Very restless	Try to remove the endotracheal tube, gastric tube, and venous access
+2	Manic anxiety	The body moves hard inability to cooperate with ventilator
+1	Disturbed anxiety	Anxiety and tension but only a slight body movement
0	Alert but quiet	Wake up the natural state
−1	Sleepy	Not fully awake, but can stay awake for more than 10 seconds
−2	Mild sedation	Unable to remain awake for more than 10 seconds
−3	Moderate sedation	Responses to sound
−4	Severe sedation	Responses to body stimuli
−5	Stupor	No response to sound or body stimulation

### Ethical Considerations

This study was approved by the Medical Ethics Committee of Shunyi Maternal and Child Health Care Hospital, Beijing (IEC-B-022-V.01-A08; Multimedia Appendix 1). As this was a retrospective study, telephone communication was conducted with legal guardians of all participating children (who experienced unplanned extubation). The research purpose, risks, and data usage scope were fully explained. All guardians provided consent for data inclusion. For secondary data analysis, the original data were anonymized, and informed consent was obtained during primary data collection. Data were deidentified by removing patient names, initials, and hospital IDs. All images in the manuscript and supplementary materials were verified to contain no identifiable features of participants. No compensation was provided to the participants. The composition and working procedures of this ethics committee complied with the principles of good clinical practice and relevant national laws and regulations.

## Results

### Demographic Risk Stratification

The demographic characteristics are provided in Table 2. Age-dependent vulnerability showed a descending gradient: infants or toddlers (≤3 years) accounted for 61.6% (8/13) of total UEX cases, preschool children (4‐6 years) for 23.1% (3/13), and school-aged children (>6 years) for 15.3% (2/13) UEX cases. Gender disparity was pronounced, with men exhibiting 2.3-fold higher risk than women (10/13, 76.9% vs 3/13, 23.1%).

**Table 2.** Pediatric patients with indwelling catheters and UEX^a^ occurrences.

Characteristics	Patients (N=13), n (%)
Gender	
Male	10 (76.9)
Female	3 (23.1)
Age (years)	
≤1	4 (30.8)
1‐3	4 (30.8)
4-6	3 (23.1)
7-16	2 (15.4)
Clinical Features	
Restraints used	
Yes	3 (23.1)
No	10 (76.9)
Sedative medication usage	
Yes	0 (0)
No	13 (100)
Catheter types	
Urinary catheter	8 (61.5)
Gastric tube	3 (23.1)
Drainage tube	2 (15.4)
PICC^b^/CVC^c^	0 (0)
UEX occurrence details	
Patient condition	
Quiet	4 (30.8)
Restless/crying	9 (69.2)
Awake	13 (100)
Comatose	0 (0)
Nurse presence	
At bedside	1 (7.7)
Not at bedside	12 (92.3)
Time period	
Morning awakening	4 (30.7)
Before/after meals	2 (15.4)
Morning/afternoon treatment	4 (30.8)
Noon/night rest	3 (23.1)
Timing post-catheter use (hours)	
≤6	2 (15.4)
24‐48	4 (30.8)
≥48	7 (53.8)
Workday/holiday	
Business days	4 (30.8)
Holidays	9 (69.2)
Tape replacement (hours)	
≤24	11 (84.6)
≥24	2 (15.4)

a UEX: unplanned extubation.

b PICC: peripherally inserted central catheter.

c CVC: central venous catheter.

### Clinical Context of UEX Events

The data in Table 3 are derived from this study's analysis of catheterized children (N=13) in the Pediatric General Surgery department from 2018 to 2023, with intergroup comparisons performed using Student *t* test. This study identified an overall UEX rate of 6.58 per 1000 catheter-days in pediatric general surgery. Catheter-specific analysis revealed that gastric tubes demonstrated the highest incidence density (44.1 events/1000 catheter-days); urinary catheters constituted the majority of UEX events (8/13, 61.5%). The RASS scores before and after catheter placement, postoperatively, and during the peri-decannulation period are shown in Table 4.

**Table 3.** Incidence of UEX^a^ in pediatric general surgery for five types of tubing from January 2018 to December 2023.

Name of tubing	Catheter days	Number of UEX occurrences	UEX incidence rate^b^	UEX for each tubing^c^, %
Urinary catheter	1079	8	7.41	61.5
Gastric tube	68	3	44.1	23.1
PICC^d^	46	0	0	0
CVC^e^	8	0	0	0
Wound drainage tube	768	2	2.6	15.4

a UEX: unplanned extubation.

b cases / catheter days × 100%.

c UEX occurrences for each tubing type / total UEX occurrences × 100%.

d PICC: peripherally inserted central catheter.

e CVC: central venous catheter.

**Table 4.** RASS scores before and after catheter placement, postoperatively, and during peri-decannulation period in 13 pediatric patients.

Rating	Before catheter placement, n	After catheter placement, n	Within 30 minutes of returning to the room postoperatively, n	After returning to the room 2 hours postoperatively, n	After 24 hours postoperatively, n	Peri-Decannulation period, n	Percentage of decannulation scoring (n=78), n (%)
+4	0	0	0	0	0	0	0
+3	0	10	3	1	4	3	21 (26.9)
+2	2	3	2	0	1	6	14 (17.9)
+1	5	0	1	3	2	2	13 (16.7)
0	6	0	1	5	6	2	20 (25.6)
−1	0	0	3	3	0	0	6 (7.7)
−2	0	0	2	1	0	0	3 (3.8)
−3	0	0	1	0	0	0	1 (1.3)
−4	0	0	0	0	0	0	0 (0)
−5	0	0	0	0	0	0	0 (0)

a RASS: Richmond Agitation-Sedation Scale.

## Discussion

### Incidence and Patient-Related Factors of UEX

#### Comparative Incidence Analysis

Existing literature demonstrates significant variability in pediatric UEX rates across care settings. PICU-focused studies such as that by Guan et al [6], a single-center analysis of 41 cases reported UEX incidence rates of 0.43‐0.79%, with 71% occurring in infants ≤12 months [12]. In mixed surgical cohorts, Sadowski et al [7] documented a higher event rate of 6.4% (95% CI 5.1‐7.8), potentially reflecting broader inclusion criteria [13]. The general pediatric surgery cohort in this study (N=1977 catheter-days) revealed a UEX incidence density of 6.58 per 1000 catheter-days, translating to a 2.1-fold higher risk than PICU benchmarks [6] and comparable rates to mixed surgical populations [7], when standardized per insertion. This discrepancy may be attributable to: (1) procedural differences that is, prolonged postoperative immobilization versus critical care protocols; (2) surveillance intensity that is, reduced monitoring frequency in general wards versus PICUs; and (3) catheter type distribution, that is, predominance of urinary or gastric devices (84.6% of UEX events) versus respiratory tubes in ICUs.

#### Age Distribution of Patients

Consistent with developmental vulnerability patterns, our findings corroborate the inverse correlation between age and UEX susceptibility first reported by Guan et al [6]. Comparative analysis revealed that infants and toddlers (≤3 years) in our cohort demonstrated 17.2% higher UEX risk compared to the study by Ma et al (Multimedia Appendix 2) [8]. This can potentially be attributable to neurodevelopmental factors (eg, immature sensorimotor integration increasing device manipulation behaviors), cognitive limitations (ie, reduced capacity to comprehend catheter purpose, as per Piaget’s preoperational stage characteristics) [14], and anatomical constraints (eg, higher device-to-body size ratio exacerbating positional discomfort). Gender disparity analysis identified men as having a 3.3-fold higher UEX risk than women (n=10, 76.9% vs n=3, 23.1%; *P*=.046), aligning with established pediatric mobility patterns. Christov-Moore et al [15] showed that this kinetic discrepancy may interact with testosterone–mediated exploratory behavior enhancement and delayed proprioceptive development in male infants.

### Consciousness State

Emerging evidence identifies altered consciousness as a critical risk predictor for UEX in pediatric populations. Pediatric patients with critical illness requiring intensive care (ie, ICU or PICU admission) demonstrate heightened susceptibility to delirium, a condition with multifactorial etiology involving both intrinsic patient factors (disease severity, metabolic disturbances) and extrinsic environmental triggers (sensory overload, sleep disruption). This neurocognitive complication is further exacerbated by iatrogenic risks associated with suboptimal care quality, including unplanned extubation events, which may amplify physiological stress and psychological distress. Such associations underscore the need for delirium-preventive protocols tailored to high-acuity pediatric settings to mitigate adverse clinical outcomes [16,17].

Standardized neurological assessment tools demonstrate clinical utility in risk stratification:

1. Pediatric GCS [18]: It assesses three domains such as ocular response (E1-E4), verbal response (V1-V5), and motor response (M1-M6). Systematic GCS documentation reduces pediatric UEX risk by 38% (odds ratio [OR] 0.62, 95% CI 0.55‐0.71) [19].
2. Confusion assessment method (CAM) [20,21]: It is an ICU-validated delirium screening tool with specificity of 93% and sensitivity 86%. The implementation of this method decreases UEX frequency by 43.7% (risk ratio [RR] 0.58; *P*=.006).

Internationally recognized critical care guidelines uniformly recommend the RASS as the criterion-standard tool for sedation assessment in adult intensive care. The RASS demonstrates high validity in both medical and surgical ICU patients, whether ventilated or nonventilated, sedated or nonanesthetized. [22]. It is recommended by the Association of the Scientific Medical Societies in Germany S2k Consensus [23], and the Chinese Expert Consensus on Neurocritical Care Sedation [24]. The operational thresholds are as follows: (1) agitation identification: RASS scores of +2 to +4 indicate clinically significant agitation requiring intervention (emergency prehospital with sensitivity of 89%) [22], and (2) sedation protocol: maintaining RASS scores between −2 to 0 reduces UEX by 41% (RR 0.59, 95% CI 0.54‐0.66) []and ventilator-associated pneumonia by 32% (HR 0.68, *P*=.007) [25].

### Comfort of Position

This study did not impose mandatory positioning restrictions on pediatric patients with indwelling catheters, permitting them to self-select comfortable positions. However, sudden postural changes were prohibited to maintain catheter stability, with continuous monitoring ensuring an indwelling segment length ≥5 cm during mobilization [26 ]. Unplanned extubation was mechanistically linked to foreign body sensation–induced physiological stress, particularly in nasogastric intubation cases where nausea incidence increased by 54% (95% CI 46%‐62%) and abdominal distension risk, (OR 2.8, 95% CI 2.1‐3.7; *P*< .001). Abdominal distension demonstrated a significant association with UEX, with affected patients exhibiting a 3.2-fold elevated risk (HR 3.2, 95% CI 2.1‐4.9; *P*=.002) compared to nondistended counterparts [27].

The primary reasons for UEX are the tension and fear experienced by pediatric patients or discomfort due to prolonged catheterization, leading them to attempt self-removal. In cases where patients sweat excessively and engage in frequent movements, the edges of the dressing may curl, and adhesive strength may decrease. Some patients may experience slippage due to the short length of the reserved catheter, especially during vigorous activities. For patients who can freely change positions and cooperate well with medical staff, it is crucial to provide specific instructions on precautions when changing positions and the placement of drainage tubes while getting up or lying down to prevent UEX.

### Distribution of High-Risk Time for UEX Occurrence

The incidence of UEX during holidays and morning hours accounts for 69.2% (n=9) and 30.7% (n=4) of the total UEX occurrence rate, respectively; on workdays, the percentage is 30.8% (n=4). For other time periods, the distribution was 15.4% (n=2) and 7.7% (n=1), respectively, indicating that holidays and the morning period (6 AM-8 AM) pose a high-risk time for UEX. Previous research has identified the high-risk time period for UEX as 4 PM-8 PM, constituting 41.2% (95% CI 38.5%‐44.0%) of occurrences. The subsequent periods from 12 AM-4 PM constitute 18.7% (95% CI 16.2%‐21.3%) [28].

### Analysis of Nursing Staff Allocation and UEX Prevention Mechanisms

Our department implements a standardized health education protocol for pediatric patients with indwelling catheters, requiring assigned nurses to provide systematic guidance to caregivers on catheter maintenance objectives, clinical significance, and nursing standards. This approach aims to reduce human–related UEX risks through enhanced caregiver awareness. Daily ward round evaluations indicate that, while parents can recall basic catheter care protocols, significant knowledge gaps persist regarding critical aspects such as postural management and recognition of abnormal signs. Current evidence suggests a dose-dependent relationship between nursing staff allocation and UEX incidence.

In critical care units, a nurse-to-patient ratio (5.00; 95%CI 2.64-7.99) increased the risk of UEX [29]. This indicates that the nurse-to-patient ratio has a direct impact on preventing UEX [30].

Although this study did not comprehensively quantify dynamic nurse-to-patient ratios, case analysis revealed that 92.3% (n=12) of UEX incidents occurred during nurse absences from the bedside, highlighting the current reliance on collaborative caregiver-staff supervision for catheter safety. Notably, our nursing team’s average clinical experience of five years has ruled out technical operational errors as primary contributors to UEX, further emphasizing the necessity for physical preventive measures (eg, optimization of restraint devices) and sustained bedside monitoring. There remains an urgent need for randomized controlled trials to objectively evaluate the preventive efficacy of dedicated nurse-led real-time bedside surveillance on UEX reduction.

### Systemic Deficiencies in Clinical Protocols Contributing to UEX

This study identified procedural deviations in medical or nursing operations as causative factors in 30.8% (n=4) of UEX incidents. Detailed root cause analysis revealed three cases of urinary catheter dislodgement attributable to balloon inflation errors (two instances involved insufficient air volume). Standard balloon inflation volume for urinary catheters is 5‐10 mL; however, pediatric patients require weight-based adjustment (0.5‐1 mL/kg, with a maximum volume not exceeding 10 mL) [31]. One case exhibited complete omission of balloon inflation. Additionally, one wound drainage tube displacement resulted from combined mechanical failures— loose fixation sutures and inadequate adhesive securement—culminating in device migration during patient ambulation.

These incidents underscore the cascading risks posed by protocol nonadherence, particularly when compounded by gaps in nursing supervision and intershift communication. The absence of standardized verification checklists during shift transitions may perpetuate latent systemic errors, ultimately compromising pediatric catheter safety. To mitigate these risks, we recommend the implementation of double-signature catheter integrity checks during shift handovers, mandatory simulation training on device-specific securement protocols (eg, ENFit compliant enteral tubes) [32].

Our analysis revealed distinct patterns in fixation efficacy: 61.5% (8/13) of UEX cases involved single-anchor fixation using hypoallergenic paper tape with an Ω-shaped cutaneous application, while 38.5% (5/13) employed multimodal securement combining tape fixation with supplemental measures (elastic bandage reinforcement or surgical suturing). The Ω technique’s biomechanical advantage lies in its motion-adaptive design—dual tape placements at strategic anatomical sites compensate for pediatric patients’ unpredictable movement ranges, thereby enhancing device stability.

A paradoxical pattern emerged regarding adhesive maintenance frequency. High-frequency replacement (<24 hours) was associated with 84.6% (n=11) of UEX incidents, whereas standard replacement (≥24 hours) was observed with only 15.4% (n=2) patients.

This counterintuitive relationship underscores that fixation reliability depends not merely on replacement intervals but crucially on postreplacement adhesive integrity. The probability of UEX detachment increases by 30% (9/30) when using tape to fix it improperly [33].

The interplay between securement methodology and material performance is further evidenced by 65% of fixation-related displacements attributable to suboptimal device-skin interface management [21]. Adopting multiple methods led to greater reduction in UEX rates than those using a single measure [34].

### Controversies in Physical Restraint Utilization for UEX Prevention

Our data revealed a 76.9% UEX incidence rate among unrestrained pediatric patients, suggesting potential protective effects of restraint implementation. This finding aligns with the outcomes of a prospective cohort study, suggesting that standardized limb restraint protocols, when properly implemented, demonstrate significant clinical value in preventing critical safety incidents including UEX and inpatient falls among high-risk populations [35]. However, international studies indicate that physical restraints are applied in 47-67% of unplanned extubation cases; however, there remains ongoing debate regarding their efficacy in preventing such incidents [36]. Current evidence suggests that restraint protocols should be dynamically adjusted based on the patient's actual clinical status, with timely and appropriate application guided by specific behavioral manifestations such as calm or agitated states [37].

Current systematic reviews conclude insufficient evidence (grade certainty: low) to support universal restraint protocols. Our findings underscore the necessity for (1) dynamic risk stratification: differentiating restraint indications between sedated (RASS −2 to 0) and agitated (RASS +1 to +3) states and (2) alternative strategies: implementing sensor-embedded alarms or distraction therapies as restraint-sparing interventions.

The absence of high-quality randomized controlled trials comparing restraint-based versus restraint-free protocols in pediatric surgical populations remains a critical knowledge gap requiring urgent investigation.

### Sedative Utilization Paradigm

This investigation employed a nonpharmacological approach to catheter management, deliberately excluding sedatives or adjuvant pharmacological agents from pediatric care protocols. Current evidence remains inconclusive regarding the prophylactic efficacy of sedation in preventing UEX, as no controlled comparative trials have established definitive causal relationships. Nevertheless, observational data from a cohort of 43 patients revealed substantial divergence in UEX incidence between nonsedated (21/43, 49%) patients and appropriately sedated groups (10/43, 23%) [38]. However, relevant studies [6] have found that among the 41 pediatric cases studied, the incidence of unplanned extubation (UEX) was 51.2% in patients without sedative use, while the rate decreased to 24.4% in those with appropriate sedation. Inadequate or absent sedation has been identified as a primary risk factor for UEX [. This underscores the imperative for protocol-driven sedation titration, ideally maintaining RASS scores between −1 and 0 during invasive device retention periods. For children using sedatives, studies have shown that the proper and adequate use of sedatives can reduce unplanned UEX events [38].[ This study did not include data on sedative use and therefore does not elaborate on the relationship between sedative use and UEX in children.

### Implementing Nursing Strategies to Reduce UEX Incidence

#### High-Risk Population Management

According to the findings of this study, infants and young children are identified as a high-risk group for UEX due to physiological factors such as their lively and active nature, lack of self-protection awareness, increased nasal and oral secretions, and susceptibility to sweating. To mitigate these risks, a tiered monitoring protocol, enhanced surveillance system was implemented. A structured rounding protocol mandated 15-minute interval checks during high-risk circadian phases (6 AM-10 PM), documented through standardized bedside monitoring protocols. Each assessment encompassed neurological status (AVPU scale scoring as shown in Multimedia Appendix 3), vital sign stability (HR variability <15%), catheter integrity metrics (migration distance <2cm; securement device adhesion >80% per Infusion therapy standards of practice [39], particularly when the childrens’ condition is unstable or they exhibit restlessness. Additionally, bedside safety measures should be implemented. Nursing staff should assist and guide parents in caring for the endotracheal tube, emphasizing key points such as the purpose and significance of catheter retention. Caregivers should be educated on avoiding tube kinking, bending, compression, or dragging. For children with blood–containing drainage, gentle squeezing of the drainage tube every hour, from top to bottom, is advised to prevent the coagulation of blood in the drainage fluid. Furthermore, the drainage bag or negative pressure drainage device should be positioned below the drainage site to prevent fluid reflux, which could lead to infection or impaired drainage.

#### Neurological Assessment Protocol

Precise evaluation of consciousness levels constitutes the cornerstone of pediatric critical care, requiring differentiation across six distinct neurological states: alertness, somnolence, obtundation, stupor, coma, and encephalopathic presentations (confusion or delirium). Validated pediatric-specific assessment instruments should be systematically implemented as follows:

- Pediatric GCS [40]: (1) ocular response: scored E1-E4 (no eye opening to spontaneous tracking); (2) verbal response: graded V1-V5 (no vocalization to oriented speech); and (3) motor response: ranked M1-M6 (no movement to purposeful obedience). The use of trained nurses for outcome assessment improved the reliability of the results [36].

- Confusion Assessment Method for ICU (CAM-ICU): Implementation of these standardized tools reduces diagnostic errors by 42% (OR=0.57, 95% CI 0.50‐0.65) compared to subjective clinical judgment alone [41]. Documentation should be aligned to the AVPU (Alert-Voice-Pain-Unresponsive) framework for rapid deterioration detection.

### Prophylactic Catheter Management Protocol

This study indicates that infants and young children constitute a high-risk group for unplanned extubation in pediatric populations due to physiological predispositions, including heightened activity levels, underdeveloped self-protection awareness, excessive oral/nasal secretions, and increased perspiration tendencies. Therefore, implementing optimized nursing protocols such as rotational fixation is recommended[42], which has demonstrated both significant reductions in unplanned extubation rates and superior outcomes in skin integrity assessment scores.

Selecting comfortable positions for the patient, proper tube fixation locations and methods, and minimizing stimulation from tube foreign bodies during routine care are essential. Sudden changes in position should be avoided to prevent UEX.

The observed temporal clustering of UEX incidents—peaking during holiday periods (n=9, 69.2%) and early morning hours (n=4, 30.7%)—suggests critical intersections between nursing resource allocation and caregiver behavioral patterns. Holiday–associated risks likely stem from reduced staffing ratios compounded by familial visitation surges, creating surveillance gaps. Morning vulnerability windows (6 AM-8 PM) may reflect attentional diversion as caregivers prioritize hygiene and nutrition tasks over device monitoring, particularly during post-anesthesia recovery phases when pediatric agitation peaks.

The relationship between quantitative disease severity scoring and nursing levels has been further clarified, providing a scientific and objective basis for clinical condition assessment. This ultimately ensures alignment between disease severity and corresponding care intensity. Recommendations include shortening ward round intervals to 30 minutes during high-risk periods and reinforcing catheter fixation protocols for the second-day care [43].

The current lack of consensus-based guidelines regarding the influence of nurse staffing parameters on adverse nursing outcomes reflects the multifactorial determinants of clinical practice, including nurses' clinical experience, professional competency, educational background, patient acuity levels, and interdepartmental variations. Given these complexities, these confounding variables were deliberately controlled for in the methodological design of this investigation.

### Strategic Staffing Allocation for UEX Risk Mitigation

The current evidence base fails to demonstrate a definitive linear correlation between hospital nursing workforce configurations and measurable patient safety outcomes. Methodological limitations in evidence appraisal reveal that the incorporation of specialized nursing personnel shows no statistically significant variation in patient mortality rates (*P*>0.05), notwithstanding these organizational interventions.

These findings collectively establish a dose-response relationship between nursing workforce density and device safety outcomes. High-acuity units demand proactive strategies including (1) implementation of dynamic staffing models that adjust real-time to patient deterioration alerts; (2) mandatory competency training on pediatric-specific restraint protocols and agitation recognition; and (3) structured family education programs using visual aids and multilingual resources to bridge health literacy gaps.

For caregivers with limited medical comprehension, iterative reinforcement through nurse-led daily demonstrations (eg, proper limb positioning during diaper changes) proves more effective than conventional verbal instructions alone.

### Optimizing Adhesive Management for Enhanced Catheter Securement

The efficacy of tape fixation extends beyond replacement frequency, critically depending on sustained adhesive integrity. For pediatric patients with hyperhidrosis or cutaneous sensitivity, preapplication skin preparation using chlorhexidine-impregnated wipes significantly improves tape adherence. Applying reinforced adhesive tape within 24 hours or combining with skin barrier products can synergistically enhance the securing effect of medical tape.

This evidence underscores a dual management imperative: (1) proactive maintenance by implementing circadian-aligned replacement schedules to prevent adhesive degradation during high-risk nocturnal agitation periods and (2) contextual adaptation by using sweat-resistant hydrocolloid tapes for tropical climates or febrile patients, coupled with twice-daily skin integrity assessments.

Thus, competency-based training modules emphasizing 45° angle tape placement and tension-free smoothing should be mandated as part of nursing credentialing programs.

### Restraint Use in UEX Prevention

While international guidelines caution against routine restraint use due to ethical and complication concerns, our data advocate for selective immobilization protocols targeting high-risk subgroups: (1) developmental vulnerability: infants or toddlers with immature impulse control (≤3 years: n=8, 61.6% of patients) and (2) pharmacological profile: nonsedated patients exhibiting RASS ≥+2 agitation scores (44.8% of incidents).

When implementing restraints, a structured safety bundle should be mandated: (1) time-limited application with 4-hour intervals with documented neurovascular assessments, and (2) positional rotation to minimize pressure points through scheduled lateral or prone positioning.

### Summary

Unplanned extubation serves as a sentinel event reflecting systemic vulnerabilities in pediatric airway safety and nursing care quality. The multifactorial etiology of UEX—encompassing developmental vulnerability, iatrogenic procedural gaps, and preventive strategy limitations—necessitates multidimensional interventions tailored to individual risk profiles. Such an approach not only reduces immediate mechanical failures but also addresses the cognitive-behavioral determinants of device interference, particularly in high-risk subgroups such as nonsedated toddlers (RASS ≥+2) with restricted comprehension capacity.

### Study Limitations

This exploratory analysis has several methodological constraints. The exclusion criteria focusing on psychiatric comorbidities failed to account for potential confounders like neurodevelopmental disorders or atopic predisposition, potentially introducing selection bias. With only 13 (76.9%) male participants, the small sample size limits generalizability and risks overestimating UEX susceptibility in infants (61.6%) while obscuring school-age children’s risk profiles. As a single-center retrospective review lacking control groups, this study could not establish causal relationships between nursing strategies and outcomes. Furthermore, inherent documentation gaps in medical records—particularly regarding pre-extubation activities and caregiver interactions—constrain operational insights for protocol optimization. Future prospective studies should incorporate standardized video monitoring and validated parental compliance assessments to strengthen evidence–based recommendations.

## Supplementary material

10.2196/71307 Multimedia Appendix 1 Ethical review and approval form for scientific research projects

10.2196/71307 Multimedia Appendix 2 Comparison of our study cohort with study by Ma et al [8]

10.2196/71307 Multimedia Appendix 3 AVPU (Alert-Voice-Pain-Unresponsive) scoring system

## Abbreviations

GCS: Glasgow Coma Scale
ICU: intensive care unit
PICU: pediatric intensive care unit
RASS: Richmond Agitation-Sedation Scale
UEX: unplanned extubation
