# Supplementary material for: Analysis of Risk Factors and Nursing Strategies for Unplanned Extubation in Children: Retrospective Cohort Study
Source: JMIR Nurs. 2025 Jun 10;8:e71307. doi: 10.2196/71307 (PMC12172804; doi:10.2196/71307)
Supplement: Multimedia Appendix 1 [file nursing-v8-e71307-s001.pdf]

Annex 8: Medical Ethics Committee of Shunyi Maternal and Child Health Care Hospital, Beijing

Ethical review and approval form for scientific research projects

(IEC-B-022-V.01-A08)

Declaration:
The composition and working procedures ofthis ethics committee comply with the principles of GCP and relevant national laws and regulations.

Ethics (fast/Meeting) reviewnumber: 2023-010-1

Project Title	Analysis of factors of unplanned extubation in children and implementation of nursing strategies					
Pricipal Investigator	Name	Xuefeng Han	Department	General Surgery	Professional Title	Nurse-in-Charge
Relevant clinical research experience	☑ Yes ☐ NO			Project timeline: January 2023-December 2023		

Submit review materials:		
1.Research Protocol	Version number: V1.0	Version date: 2023.1.1
2.Informed Consent Form	Version number: V1.0	Version date: 2023.1.1

Type of Review		☐ Fast review ☑ Conference review	
Rapid review	Signature of the Chairperson		
	Review results	☐ Submit the agreement to the meeting for review	☐ Submit it for review at the meeting by consensus

Review by the Conference	Attendance	There should be 15 people, 15 people actually showed up, 0 people were excused and 15 people voted
	Voting Results	Agreed 15, agreed with necessary amendments 0, reviewed with necessary amendments 0, disagreed 0, terminated or suspended approved projects 0.
Tracking review frequency<br>(From the date of project approval)		☐3 months ☐6 months ☑12 months other
Ethical approval validity period<br>(From the date of project approval)		☐6 months ☐9 months ☐12 months ☑other

Conclusions of the review:

According to the relevant ethical review principles, this medical ethics committee has reviewed and agreed to carry out scientific research on the project

Signature of the Chairman/Deputy Chairman:

Date: March 2023

Shunyi Maternal and Child Health Hospital, Beijing

Medical Ethics Committee (with seal)

Medical Ethics Committee of Shunyi Maternal and Child Health Care Hospital, Beijing
No.1 Shunkang Road, Shunyi District, Beijing
TEL:010-59612575 FAX:010-59612575 E-mail:SYFYIEC@163.com
