# Supplementary material for: Analysis of Risk Factors and Nursing Strategies for Unplanned Extubation in Children: Retrospective Cohort Study
Source: JMIR Nurs. 2025 Jun 10;8:e71307. doi: 10.2196/71307 (PMC12172804; doi:10.2196/71307)
Supplement: Multimedia Appendix 2 [file nursing-v8-e71307-s002.docx]

| Study Cohort | Infant (%) | Toddler (%) | Preschooler (%) | School-Age (%) |
| --- | --- | --- | --- | --- |
| Ma et al. (N=221)  [8] | 52.5 | 14.9 | 5.0 | 27.6 |
| Current Study (N=13) | 61.6 | - | 23.1 | 15.3 |
