# Supplementary material for: Analysis of Risk Factors and Nursing Strategies for Unplanned Extubation in Children: Retrospective Cohort Study
Source: JMIR Nurs. 2025 Jun 10;8:e71307. doi: 10.2196/71307 (PMC12172804; doi:10.2196/71307)
Supplement: Multimedia Appendix 3 [file nursing-v8-e71307-s003.docx]

| Level | Response Description |
| --- | --- |
| A-Alert | The child is awake, interacts appropriately with the environment, and responds to verbal communication. |
| V-Voice | The child responds to verbal stimuli (e.g., shouting or commands) but may be confused or disoriented. |
| P-Pain | The child reacts only to painful stimuli (e.g., trapezius squeeze or nailbed pressure) with purposeful or nonpurposeive movement. |
| U-Unresponsive | The child does not respond to voice or pain. Immediate airway and neurological interventions are required. |
